# Resident Medical Officership in the Future

**Published:** 1912-09-07

**Authors:** 


### RESIDENT MEDICAL OFFICERSHIP IN THE FUTURE.

### The Advantages and Disadvantages of such a Career.

There can be no two opinions that to substitute institutional medical treatment for private practice has been the trend of things ever since the year 1845. Under the provisions of the great English Lunacy Act, passed through the exertions of the then Lord Shaftesbury, asylums were erected in every county of this country. Even before that, institutions for care of the sick were rapidly growing in number and scope, and gaining a high position in public estimation. The Metropolitan Board fever hospitals gave, in the last quarter of the nineteenth century, a strong impetus to this movement, as, too, in the last twelve years or so have public sanatoriums for consumption. What with establishments of the kinds already mentioned, epileptic colonies, Poor-Law infirmaries, hospitals for incurables, and so forth, there is, if not a very large, at any rate an extending field of work for those who hope for resident charge of institutional patients.

### The Decline of General Practice

The advantages and disadvantages of such a career have been often discussed before, but not in the light of recent events. One now sees general practice of the old kind threatened on many sides. For one thing, it has come to demand far too much of a man. The forward march of medicine has caused the clinching of a diagnosis or the efficacy of treatment to depend upon the services of specialists. There are practitioners—just a few—who can take a Wassermann reaction in the afternoon, do version at night, and gastro-jejunostomy the next morning, separate bronchial catarrhs from incipient phthisis correctly and consistently (would that the ordinary consultant had half their native ability!), but they are necessarily very rare. Hence plenty are ready to vacate practices for a well-paid resident post. As for the public gain, that is most obvious. To begin with, the doctor is where he is wanted to be—on the spot. Then, in place of the upsetting of numerous households with amateurish efforts at preparation for illness, the business is taken over, to be carried out much more economically and efficiently, by one central depôt. Further, the wealth of homogeneous clinical material enables exact experiment to be made on various kinds of treatment, the correct appraising of which demands prolonged and extensive trial. A weekly or monthly visit of friends gives the patient quite as much family sympathy as is wholesome; in a small middle-class household, or elsewhere for the matter of that, an invalid can soon become a vampire. Lastly, our national insularity and unsociableness are usefully repressed. This is why the fever hospital is supplanting the top back bedroom with its carbolic sheet; the open-air school, the useless "bottle of medicine;" the home for incurables, the lonely mattress-grave; and sanatoriums and tuberculosis dispensaries, the out-patient department.

Really good working of such institutions, it soon becomes evident to all concerned, depends upon the character of its resident head. Safeguard the management too much, exclude by strict regulations every chance of important duties being neglected, and you destroy—as Sir Frederick Treves, with characteristic common sense, pointed out in the case of the old system of the Army Medical Department—initiative and interest in work and efficiency. Further, an institution, the primary object of which is medical, should, in all its details, be directed in deference to medical opinion. In all details, therefore, administrative as well as medical, the resident medical officer of the future must be left a free hand.

He must know that wood beats earthenware for laundry troughs, as well as the use of ophthalmoscope or autoclave. To technical accomplishment the resident head of the establishment must join the ability to give definite orders on every point that crops up, and to get such orders carried out smartly. In the life of the institutional resident there need be no banal social calls, no coaching of students for pocket money, no struggle to get beds for his cases, no travelling to and fro, none of the countless conventional leakages of time the junior metropolitan consultant has to suffer: while in place of toiling over the wide field of surgery, or the now narrower but highly intensive one of internal medicine, his attention is concentrated upon one class of case, or even upon one disease. If biologists have to make themselves conversant with the practice of stock fanciers and nurserymen, doing so without any loss of prestige, a little versatility should not be fatal to medical reputation. Mental hospital superintendents are now recognised to be the authorities in psychiatry, and ere long similar recognition will be extended to other resident institutional special workers. Not but what it is necessary to have picked men, but the selection should be made after the method of trial and error. There should be no certainty of tenure aforehand. Academic successes alone must be thought less of. But where intellectual and administrative ability, both freely and disinterestedly given, are found existing together, it may be taken as certain that here is a man to keep and to promote and to listen to. He will not need to write redundant text-books in imperfectly clear English, or to run after Lady Bountifuls or bishops. It is a type which further offers to the individual aspirant positions which do not compare unfavourably with the desperately-won or chance-bestowed prizes of competitive private practice, and to the resident officer insecurity of tenure is a stimulus and not a menace. The worst hectoring of committees, wherever it now exists, to a strong man is not so harrowing as the professional and social "claims" which often demand so undue a service in private practice. In institutional work doctoring becomes more of a science and less of an art, while the versatility which the better posts exact is a more valuable personal asset than the dexterity of the specialist or the address of the popular practitioner.

